# Combined associations of physical activity and sedentary behavior with all-cause and cardiovascular mortality in hypertensive adults: A cohort study using NHANES data (2007–2018)

**DOI:** 10.1097/MD.0000000000047167

**Published:** 2026-01-23

**Authors:** Wenjing Zhang, Yihang Du, Zizhen Chen, Mengxue Wang, Ting Zhao, Yuanhui Hu

**Affiliations:** a Guang'anmen Hospital, China Academy of Chinese Medical Sciences, Beijing, China.

**Keywords:** all-cause mortality, cardiovascular mortality, hypertension, physical activity (PA), sedentary behavior (SB)

## Abstract

With rising sedentary behavior (SB) and reduced physical activity (PA) levels, hypertensive adults face an escalated risk of mortality. The individual impacts of PA and SB on mortality are well-documented, yet their combined effects on survival outcomes in this population remain underexplored. This study investigates the combined associations of PA and SB with all-cause and cardiovascular mortality risks in hypertensive U.S. adults. This cohort study utilized data from the National Health and Nutrition Examination Survey (2007–2018) with mortality follow-up through December 31, 2019. PA and SB were assessed using the Global Physical Activity Questionnaire. Mortality status and cause of death were ascertained by linkage to the National Death Index. Kaplan–Meier curves and the Cox proportional hazard model were used to evaluate the associations between separate and joint prognostic effects of PA and SB with mortality outcomes among hypertensive adults. Hypertensive individuals who engaged in short-term sitting (≤6 hours/day) and active physical activity (PA ≥ 600 metabolic equivalent [MET]-minute/week) exhibited the lowest all-cause mortality risk (hazard ratio = 0.42, 95% CI: 0.36–0.48, *P* < .001). Similarly, those with short-term sitting (≤6 hours/day) and insufficiently active PA (0 < PA < 600 MET-minute/week) showed the lowest cardiovascular mortality risk (hazard ratio = 0.36, 95% CI: 0.24–0.53, *P* < .001). Conversely, hypertensive adults with long-term sitting (>6 hours/day) and no physical activity (PA = 0 MET-minute/week) faced the highest risks of all-cause and cardiovascular mortality. Kaplan–Meier survival analysis further confirmed that hypertensive patients with short-term sitting and active PA had significantly higher overall survival probabilities compared to other groups. Our study highlights that the combination of active PA and short-term SB was strongly associated with reduced mortality risk of hypertensive adults. Our findings might help to refine the lifestyle intervention recommendations for this population.

## 1. Introduction

Hypertension is a prevalent chronic condition globally and a significant driver of cardiovascular disease incidence and mortality. The Global Burden of Disease (GBD) report highlights a steady rise in hypertension prevalence, especially among aging, increasingly sedentary populations.^[1]^ The 2024 Heart Disease and Stroke Statistics report estimates that approximately 47% of U.S. adults (around 122.4 million people) have hypertension, with hypertension-related cardiovascular diseases contributing to nearly 10 million global deaths annually.^[2]^ Hypertension accelerates arterial sclerosis and endothelial dysfunction, elevating risks for stroke, myocardial infarction, and heart failure.^[3–5]^ Thus, its prevention and management remain critical global health priorities. Despite advancements in pharmacological treatments, mortality and cardiovascular events in hypertensive individuals remain high, revealing the limitations of medication alone.^[6,7]^

Physical activity (PA) has demonstrated protective effects against chronic diseases and cardiovascular disease, with evidence indicating that regular, moderate PA reduces the risk of early cardiovascular–kidney–metabolic syndrome,^[8]^ cardiovascular disease mortality, and all-cause mortality.^[9]^ For hypertensive individuals, moderate exercise helps reduce blood pressure (BP), improves lipid profiles, and enhances vascular elasticity, thereby slowing disease progression.^[10–12]^ Consequently, international health guidelines emphasize PA as essential in lifestyle interventions for hypertensive patients.^[13,14]^ However, the mechanisms and potential dose–response relationship between PA and reductions in cardiovascular disease mortality risk among individuals with hypertension are not yet fully understood. Existing research offers limited insight into survival benefit thresholds across different PA levels, critical for developing personalized interventions. Conversely, sedentary behavior (SB) has become an independent mortality risk factor, particularly when PA is insufficient.^[15]^ SB is associated with low metabolic activity and conditions like obesity and metabolic syndrome, with evidence showing a strong link between extended SB and increased mortality and cardiovascular risk, even among those with higher PA levels.^[16–18]^

Research on the combined impact of PA and SB specifically on all-cause and cardiovascular mortality among hypertensive patients is limited. Most studies tend to examine PA or SB individually, often overlooking their interaction in this high-risk group.^[19–21]^ Questions remain unanswered, such as whether moderate PA mitigates SB’s adverse effects or at what PA volume mortality risks associated with SB are significantly reduced in hypertensive individuals.^[22]^ Thereby limiting the development of tailored interventions to some extent.

This study utilizes large-scale population data to examine the combined effects of PA and SB on all-cause and cardiovascular mortality among individuals with hypertension. By quantifying the nonlinear relationships among PA, SB, and survival rates, this research explores the interactive impact of PA and SB on mortality risk, aiming to provide a scientific basis for developing more precise and effective health intervention strategies for those with hypertension.

## 2. Methods

### 2.1. Study participants

**Figure 1.**
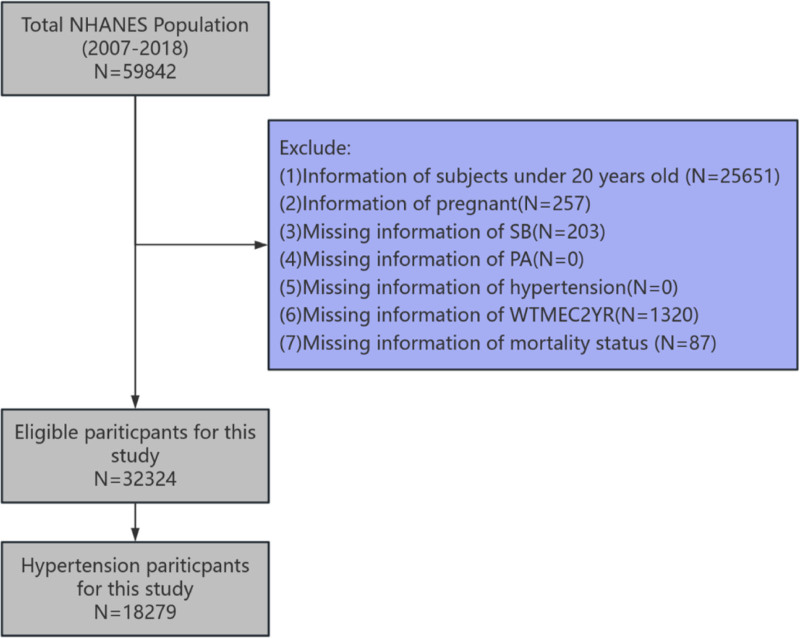
Screening process for participants.

This study utilized data from the National Health and Nutrition Examination Survey cycles 2007 to 2018. The initial population comprised 59,842 participants. We sequentially excluded participants who were under 20 years of age (N = 25,651), pregnant (N = 257), or had missing data on SB (N = 203), mortality status (N = 87), or examination weights (N = 1320). The final analytical cohort consisted of 18,279 adults with hypertension. The participant selection process is detailed in Figure 1. The study received National Center for Health Statistics Ethics Review Board approval, and all participants provided informed consent.

### 2.2. Assessment of PA and SB

The Global Physical Activity Questionnaire, widely used to monitor SB trends^[23]^ and health impacts,^[24]^ collected data on PA frequency, duration, and intensity across different domains (the detailed assessment for PA can be found on the website https://www.who.int/teams/noncommunicable-diseases/surveillance/systems-tools/physical-activity-surveillance). PA was expressed in metabolic equivalent (MET)-minutes per week for moderate-to-vigorous intensity activities, calculated according to World Health Organization guidelines. MET values corresponding to each exercise modality were obtained from the National Health and Nutrition Examination Survey. PA scores were calculated in MET-minute/week using the formula: MET value of the activity × weekly frequency × duration per session.^[25]^ The vigorous-intensity physical activity scores 8 MET; moderate-intensity physical activity, and walking or bicycling for transportation score 4 MET. Time spent in light activity and sedentary activities such as watching TV and using computer were not included in the MET calculation. According to the 2018 Physical Activity Guidelines for Americans, adults should engage in moderate-intensity PA for 150 minutes per week or vigorous-intensity activity for 75 minutes per week (all equivalent to 600 MET-minute/week).^[26]^

Based on the MET calculation formula and the PA requirements in the guidelines, we classified participants into 3 groups: inactive (PA = 0), insufficiently active (0 < PA < 600 MET-minute/week), and active (PA ≥ 600 MET-minute/week).^[27]^ Additionally, to examine the dose–response relationship between PA and mortality risk in hypertensive patients, we established the following categories for PA: 0 MET-minute/week, 0 < PA < 600, 600 ≤ PA < 2000, 2000 ≤ PA < 4000, and PA ≥ 4000 MET-minute/week, based on previous research studies.^[21,28]^ SB data were similarly obtained via a questionnaire. For the primary joint association analysis, SB was dichotomized into short-term sitting (≤6 hours/day) and long-term sitting (>6 hours/day) based on thresholds commonly used in prior literature and their clinical relevance.^[29]^ Additionally, to investigate the dose–response relationship between SB and mortality risk in hypertensive patients, sedentary time was categorized into ≤4, 4 to 8, and >8 hours per day,^[30]^ and was further divided into the following groups: >8 hours/day, 6 hours/day ≤ SB ≤ 8 hours/day, 4 hours/day ≤ SB < 6 hours/day, and SB < 4 hours/day.

### 2.3. Assessment of hypertension

Participants were defined as having hypertension if they had a systolic BP ≥ 130 mm Hg, diastolic BP ≥ 80 mm Hg, or reported current use of antihypertensive medication, consistent with the 2017 ACC/AHA guideline.^[31]^

### 2.4. Mortality determination

The mortality data were obtained from the National Death Index by the National Center for Health Statistics, with follow-up concluding on December 31, 2019. Death causes were categorized based on the Tenth Revision of the International Classification of Diseases codes (https://icd.who.int/browse10/2019/en#/IX). All-cause mortality encompassed death from any cause, while cardiovascular mortality included Tenth Revision of the International Classification of Diseases codes I00-I09, I11, I13, and I20-I51.^[19]^

### 2.5. Covariate assessment

Covariates included sociodemographic factors, lifestyle behaviors, and health conditions. Sociodemographic variables were sex (female, male), age (<60, ≥60), race/ethnicity (Mexican American, other Hispanic, non-Hispanic White, non-Hispanic Black, other/multiracial), marital status (married, never married, living with partner, or other), education level (high school or below, some college, college graduate or above), and household income (high, medium, low). Lifestyle factors included alcohol intake (nondrinker, 1–5 drinks/month, 5–10 drinks/month, 10+ drinks/month) and smoking status (never, former, current). Body mass index (BMI) was classified into underweight (<18.5), normal (18.5–<25), overweight (25–<30), and obese (≥30). Medical history included diabetes (yes/no), cancer (yes/no), and cardiovascular disease (CVD) history (yes/no). Cancer and cardiovascular history was gathered via computer-assisted interviews, and diabetes was defined as fasting plasma glucose ≥ 7.0 mmol/L, hemoglobin A1c ≥ 6.5%, current antidiabetic medication use, or a physician’s diagnosis.^[32]^

### 2.6. Statistical methods

All analyses employed weighted, clustered, and stratified methods to ensure national representativeness. Sample sizes and weighted percentages were calculated for sociodemographic, lifestyle, health, PA, and SB variables to ensure representativeness. Descriptive statistics summarized participants’ baseline characteristics. The Kolmogorov–Smirnov test assessed normality for continuous variables. Non-normal variables were presented as medians with interquartile ranges, and categorical variables as percentages. Baseline differences were examined using the Mann–Whitney *U* and Chi-square tests. Kaplan–Meier curves illustrated survival differences across groups, with log-rank tests for statistical comparison. According to previous literature,^[28]^ a sedentary threshold of 6 hours/day and a PA threshold of 600 MET-minute/week were set for further analyses. Participants were then divided into 6 groups: SS-INA (short-term sitting, ≤6 hours/day and inactive, PA = 0), SS-IA (short-term sitting, ≤6 hours/day and insufficiently active, 0 < PA < 600 MET-minute/week), SS-AC (short-term sitting, ≤6 hours/day and active, PA ≥ 600 MET-minute/week), LS-INA (long-term sitting, >6 hours/day and inactive, PA = 0), LS-IA (long-term sitting, >6 hours/day and insufficiently active, 0 < PA < 600 MET-minute/week), and LS-AC (long-term sitting, >6 hours/day and active, PA ≥ 600 MET-minute/week). This categorization enables a detailed exploration of how these factors jointly influence the study outcomes. Several Cox proportional hazards models were developed to examine the relationships between PA, SB, and both all-cause and cardiovascular mortality. Model 1 was unadjusted, while Model 2 accounted for age, sex, and race; and Model 3 further accounted marital status, education attainment, family income, alcohol intake, smoking, BMI, diabetes, and CVD history (congestive heart failure, coronary heart disease, angina pectoris, and stroke). Variables with missing data were imputed using multiple imputation methods. Last, sensitivity analysis was conducted by excluding participants who died during the first 24 months of follow-up to minimize the potential impact of reverse causation. All analyses were performed using R software (version 4.3.1; R Foundation for Statistical Computing, Vienna, Austria, https://www.R-project.org/).

## 3. Results

### 3.1. Participant baseline characteristics

The study included a weighted total of 18,279 participants with hypertension (weighted population: 114,063,170), with a median follow-up time of 6.17 years (interquartile range: 3.42–9.33years). Participants were categorized into 6 subgroups based on SB and PA levels, as shown in Table 1. The results indicated that the overall sample had a nearly balanced gender distribution (52% men and 48% women), with a median age of 55 years and a predominance of non-Hispanic White individuals (68%). Forty-two percent of the participants had a high school education or less, and middle-income households accounted for the highest proportion (48%), the group comparisons revealed significant differences (*P* < .001). The proportion of women was higher in the LS-INA (59%) and SS-INA (61%) groups, while men were dominant in the LS-AC (61%) and SS-AC (59%) groups. In terms of age distribution, the LS-AC group was the youngest (median age: 52 years), whereas the LS-INA and SS-INA groups were the oldest (median age: 61 years). Socioeconomic characteristics further showed that the LS-AC group had the highest proportion of high-income individuals (36%) and those with college graduate or above (39%). In contrast, the SS-INA group had the highest proportions of low-income individuals (33%) and nondrinkers (37%). With regard to health indicators, the LS-INA group demonstrated the highest rates of obesity (58%), diabetes (32%), and CVD, with a median BMI of 31. In terms of mortality, the LS-INA group exhibited the highest all-cause mortality rate (23%) and cardiovascular mortality rate (6.5%), while the LS-AC group had the lowest rates, with all-cause mortality at 5.6% and cardiovascular mortality at just 1.5%. Tables S1 and S2, Supplemental Digital Content, https://links.lww.com/MD/R179 categorized participants based on different levels of SB and PA, respectively. The analysis revealed that groups with longer sedentary time and lower activity levels exhibited significantly higher rates of all-cause mortality and cardiovascular mortality at baseline.

**Table 1:** Baseline characteristics of NHANES individuals with hypertension.

Characteristic	Overall, (n = 18,279) weighted%:100.0	SB and PA levels						*P*
		LS-INA, (n = 2746) weighted%:15.0	SS-INA, (n = 3152) weighted%:17.2	LS-AC, (n = 2980) weighted%:16.3	SS-AC, (n = 6760) weighted%:37.0	LS-IA, (n = 1147) weighted%:6.3	SS-IA, (n = 1494) weighted%:8.2	
Sex								<.001
Female	8798 (48%)	1545 (59%)	1867 (61%)	1163 (39%)	2752 (41%)	585 (51%)	886 (61%)	
Male	9481 (52%)	1201 (41%)	1285 (39%)	1817 (61%)	4008 (59%)	562 (49%)	608 (39%)	
Age (yr)	55 (43, 67)	61 (50, 74)	61 (50, 73)	52 (39, 61)	53 (41, 64)	54 (44, 65)	59 (47, 70)	<.001
Age group								<.001
<60 yr	9186 (60%)	1005 (46%)	1164 (46%)	1897 (71%)	3857 (65%)	610 (62%)	653 (52%)	
60–69 yr	4023 (18%)	581 (20%)	830 (21%)	563 (16%)	1434 (18%)	253 (18%)	362 (19%)	
70–79 yr	2715 (12%)	488 (15%)	613 (17%)	295 (7.9%)	881 (10%)	158 (11%)	280 (17%)	
80+ yr	2355 (10.0%)	672 (19%)	545 (17%)	225 (5.5%)	588 (6.4%)	126 (8.5%)	199 (12%)	
Race								<.001
Mexican American	2395 (6.9%)	258 (4.6%)	609 (11%)	243 (4.3%)	976 (8.1%)	82 (3.6%)	227 (8.0%)	
Other Hispanic	1779 (4.9%)	221 (3.6%)	396 (7.1%)	207 (3.8%)	715 (5.5%)	80 (3.3%)	160 (5.5%)	
Non-Hispanic White	7560 (68%)	1346 (73%)	1027 (57%)	1332 (72%)	2816 (67%)	504 (71%)	535 (62%)	
Non-Hispanic Black	4570 (13%)	692 (13%)	812 (16%)	792 (12%)	1559 (12%)	328 (14%)	387 (15%)	
Other/multiracial	1975 (7.6%)	229 (6.0%)	308 (9.2%)	406 (7.6%)	694 (7.0%)	153 (8.7%)	185 (9.6%)	
Marital status								<.001
Married	9641 (57%)	1309 (53%)	1637 (55%)	1585 (57%)	3710 (59%)	595 (59%)	805 (58%)	
Never married	2269 (12%)	275 (9.6%)	291 (9.5%)	503 (16%)	888 (13%)	134 (11%)	178 (11%)	
Living with partner	1113 (6.4%)	116 (5.1%)	153 (5.2%)	206 (6.6%)	509 (7.5%)	60 (4.8%)	69 (5.7%)	
Other	5256 (24%)	1046 (33%)	1071 (30%)	686 (20%)	1653 (20%)	358 (26%)	442 (25%)	
Education attainment								<.001
High school or less	9356 (42%)	1479 (45%)	2098 (60%)	1030 (28%)	3463 (44%)	435 (29%)	851 (47%)	
Some college	5231 (32%)	777 (32%)	698 (26%)	979 (33%)	2009 (33%)	380 (35%)	388 (28%)	
College graduate or above	3692 (26%)	490 (23%)	356 (15%)	971 (39%)	1288 (23%)	332 (36%)	255 (24%)	
Family income								<.001
High income	3216 (27%)	427 (24%)	355 (16%)	778 (36%)	1185 (26%)	268 (33%)	203 (23%)	
Low income	6545 (25%)	1030 (27%)	1355 (33%)	845 (19%)	2340 (24%)	366 (22%)	609 (29%)	
Medium income	8518 (48%)	1289 (48%)	1442 (51%)	1357 (45%)	3235 (50%)	513 (45%)	682 (48%)	
Alq group								<.001
Nondrinker	5647 (24%)	973 (31%)	1326 (37%)	681 (18%)	1819 (21%)	303 (19%)	545 (29%)	
1–5 drinks/mo	8706 (48%)	1320 (50%)	1405 (45%)	1481 (48%)	3224 (48%)	580 (49%)	696 (48%)	
5–10 drinks/mo	1235 (8.3%)	125 (5.2%)	142 (5.7%)	258 (10%)	553 (9.3%)	81 (9.4%)	76 (6.8%)	
10+ drinks/mo	2691 (19%)	328 (14%)	279 (12%)	560 (24%)	1164 (21%)	183 (23%)	177 (16%)	
Smoke group								<.001
Never smoker	9537 (52%)	1331 (49%)	1730 (54%)	1599 (55%)	3471 (50%)	583 (53%)	823 (53%)	
Former smoker	5213 (29%)	890 (33%)	831 (26%)	827 (29%)	1898 (29%)	348 (29%)	419 (29%)	
Current smoker	3529 (19%)	525 (18%)	591 (19%)	554 (16%)	1391 (21%)	216 (18%)	252 (18%)	
BMI	29 (26, 34)	31 (27, 37)	29 (26, 34)	30 (26, 34)	29 (25, 33)	30 (27, 36)	29 (25, 34)	<.001
BMI group								<.001
Normal (18.5–<25)	3754 (20%)	463 (14%)	637 (20%)	571 (19%)	1541 (23%)	219 (16%)	323 (22%)	
Obese (≥30)	8371 (47%)	1477 (58%)	1423 (47%)	1441 (48%)	2809 (42%)	566 (51%)	655 (44%)	
Overweight (25–<30)	5958 (33%)	770 (27%)	1061 (32%)	940 (33%)	2350 (35%)	347 (32%)	490 (32%)	
Underweight (<18.5)	196 (1.0%)	36 (1.4%)	31 (0.9%)	28 (0.9%)	60 (0.8%)	15 (1.0%)	26 (1.9%)	
Diabetes								<.001
Yes	4828 (21%)	987 (32%)	1072 (29%)	613 (16%)	1407 (16%)	318 (23%)	431 (22%)	
No	13,451 (79%)	1759 (68%)	2080 (71%)	2367 (84%)	5353 (84%)	829 (77%)	1063 (78%)	
Congestive heart failure								<.001
Yes	991 (4.2%)	299 (8.7%)	226 (6.4%)	121 (3.1%)	217 (2.4%)	68 (4.5%)	60 (3.5%)	
No	17,288 (96%)	2447 (91%)	2926 (94%)	2859 (97%)	6543 (98%)	1079 (95%)	1434 (96%)	
Coronary heart disease								<.001
Yes	1172 (5.6%)	283 (9.2%)	241 (7.1%)	162 (4.6%)	342 (4.6%)	73 (5.0%)	71 (4.3%)	
No	17,107 (94%)	2463 (91%)	2911 (93%)	2818 (95%)	6418 (95%)	1074 (95%)	1423 (96%)	
Angina pectoris								<.001
Yes	713 (3.6%)	159 (5.9%)	156 (5.0%)	97 (2.4%)	213 (3.2%)	46 (2.8%)	42 (2.1%)	
No	17,566 (96%)	2587 (94%)	2996 (95%)	2883 (98%)	6547 (97%)	1101 (97%)	1452 (98%)	
Stroke								<.001
Yes	1124 (4.9%)	310 (9.6%)	274 (7.8%)	133 (3.2%)	258 (3.1%)	68 (3.6%)	81 (5.1%)	
No	17,155 (95%)	2436 (90%)	2878 (92%)	2847 (97%)	6502 (97%)	1079 (96%)	1413 (95%)	
Cancer								<.001
Yes	2377 (14%)	487 (18%)	432 (15%)	345 (13%)	775 (13%)	172 (17%)	166 (13%)	
No	15,902 (86%)	2259 (82%)	2720 (85%)	2635 (87%)	5985 (87%)	975 (83%)	1328 (87%)	
All-cause mortality	2620 (11%)	781 (23%)	599 (17%)	241 (5.6%)	635 (7.0%)	154 (10%)	210 (10.0%)	<.001
Cardiovascular mortality	668 (2.7%)	218 (6.5%)	143 (3.9%)	65 (1.5%)	167 (1.8%)	33 (2.1%)	42 (2.0%)	<.001
PERMTH_EXM	74 (41, 112)	66 (37, 97)	76 (38, 115)	73 (43, 106)	79 (41, 119)	71 (43, 101)	84 (44, 119)	<.001

Median (Q1, Q3); n (%). *P* < .05 was considered significant.

BMI = body mass index; LS-AC = long-term sitting, >6 hours/day and active, PA ≥ 600 MET-minute/week; LS-IA = long-term sitting, >6 hours/day and insufficiently active, 0 < PA < 600 MET-minute/week; LS-INA = long-term sitting, >6 hours/day and inactive, PA = 0; NHANES = National Health and Nutrition Examination Survey; PA = physical activity; SB = sedentary behavior; SS-AC = short-term sitting, ≤6 hours/day and active, PA ≥ 600 MET-minute/week; SS-IA = short-term sitting, ≤6 hours/day and insufficiently active, 0 < PA < 600 MET-minute/week; SS-INA = short-term sitting, ≤6 hours/day and inactive, PA = 0.

### 3.2. Relationship between PA, SB, and mortality

During a median follow-up period of 6.17 years, a total of 3288 deaths occurred, including 2620 participants who died from hypertension and 668 from cardiovascular diseases. Fully adjusted models revealed that hypertension patients with adequate physical activity (PA ≥ 4000 MET-minute/week) had the lowest all-cause mortality (hazard ratio [HR] 0.49, 95% CI: 0.41–0.59). In contrast, those with 2000 ≤ PA < 4000 MET-minute/week had the lowest cardiovascular mortality (HR 0.39, 95% CI: 0.26–0.60). Furthermore, hypertensive patients with sedentary time <4 hours per day demonstrated lower all-cause mortality (HR 0.66, 95% CI: 0.57–0.77) and cardiovascular mortality (HR 0.52, 95% CI: 0.36–0.74) compared to those with sedentary time exceeding 8 hours per day (Table 2).

**Table 2:** Association of PA and SB with all-cause, and cardiovascular mortality among individuals with hypertension.

Characteristic	Death/no.	Model 1		Model 2		Model 3	
		95% CI	*P*	95% CI	*P*	95% CI	*P*
PA							
All-cause mortality							
PA = 0	1380/5898	–		–		–	
0 < PA < 600	364/2641	0.47 (0.40–0.55)	<.001	0.58 (0.50–0.67)	<.001	0.68 (0.59–0.79)	<.001
600≤PA < 2000	446/3767	0.4 (0.34–0.46)	<.001	0.48 (0.42–0.55)	<.001	0.6 (0.52–0.69)	<.001
2000≤PA < 4000	177/2081	0.28 (0.22–0.37)	<.001	0.38 (0.31–0.48)	<.001	0.51 (0.41–0.62)	<.001
PA≥4000	253/3892	0.21 (0.18–0.26)	<.001	0.37 (0.31–0.44)	<.001	0.49 (0.41–0.59)	<.001
Cardiovascular mortality							
PA = 0	361/5898	–		–		–	
0 < PA < 600	75/2641	0.36 (0.27–0.48)	<.001	0.47 (0.36–0.62)	<.001	0.59 (0.45–0.77)	<.001
600≤PA < 2000	122/3767	0.45 (0.34–0.60)	<.001	0.56 (0.43–0.73)	<.001	0.72 (0.54–0.96)	.023
2000≤PA < 4000	41/2081	0.19 (0.13–0.29)	<.001	0.28 (0.18–0.42)	<.001	0.39 (0.26–0.60)	<.001
PA≥4000	69/3892	0.21 (0.14–0.30)	<.001	0.4 (0.28–0.57)	<.001	0.58 (0.40–0.85)	.005
SB							
All-cause mortality							
SB > 8 h/d	628/3606	–		–		–	
6 h/d ≥ SB ≤ 8 h/d	907/5422	0.95 (0.84–1.08)	.4	0.75 (0.67–0.84)	<.001	0.85 (0.74–0.96)	.012
4 h/d ≥ SB < 6 h/d	563/4386	0.69 (0.59–0.81)	<.001	0.58 (0.50–0.67)	<.001	0.72 (0.62–0.84)	<.001
SB < 4 h/d	522/4865	0.55 (0.48–0.64)	<.001	0.54 (0.47–0.61)	<.001	0.66 (0.57–0.77)	<.001
Cardiovascular mortality							
SB > 8 h/d	156/3606	–		–		–	
6 h/d ≥ SB ≤ 8 h/d	253/5422	1.05 (0.83–1.33)	.7	0.8 (0.63–1.01)	.063	0.94 (0.72–1.21)	.6
4 h/d ≥ SB < 6 h/d	153/4386	0.74 (0.55–1.01)	.056	0.61 (0.45–0.83)	.002	0.8 (0.58–1.10)	.2
SB < 4 h/d	106/4865	0.42 (0.30–0.60)	<.001	0.4 (0.29–0.56)	<.001	0.52 (0.36–0.74)	<.001

Model 1 served as the unadjusted analysis. Model 2: adjusted for age, sex, and race. Model 3: fully adjusted model, including additional covariates such as marital status, education attainment, family income, alcohol intake, smoking status, BMI, sitting time, diabetes, and CVD history (congestive heart failure, coronary heart disease, angina pectoris, and stroke). *P*-value <.05 was considered significant.

BMI = body mass index, CI = confidence interval, CVD = cardiovascular disease, HR = hazard ratio, PA = physical activity, SB = sedentary behavior.

### 3.3. Joint association of PA and SB with mortality

**Figure 2.**
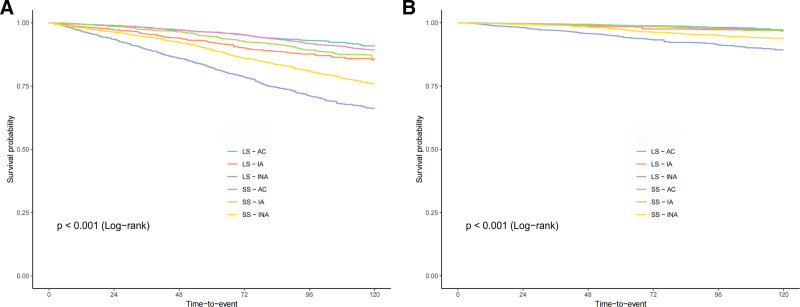
Kaplan–Meier survival curves for all-cause mortality (A) and cardiovascular mortality (B) based on PA and SB categories. LS-AC = long-term sitting, >6 hours/day and active, PA ≥ 600 MET-minute/week; LS-IA = long-term sitting, >6 hours/day and insufficiently active, 0 < PA < 600 MET-minute/week; LS-INA = long-term sitting, >6 hours/day and inactive, PA = 0; PA = physical activity; SB = sedentary behavior; SS-AC = short-term sitting, ≤6 hours/day and active, PA ≥ 600 MET-minute/week; SS-IA = short-term sitting, ≤6 hours/day and insufficiently active, 0 < PA < 600 MET-minute/week; SS-INA = short-term sitting, ≤6 hours/day and inactive, PA = 0.

As illustrated in Table 3, in the joint analysis, the combination of short-term sitting and active PA was associated with the lowest risks of all-cause and cardiovascular mortality. Specifically, compared to other groups in the fully adjusted model, hypertensive patients with short-term sitting (≤6 hours/day) and active PA (PA ≥ 600 MET-minute/week) and exhibited the lowest all-cause mortality risk (HR = 0.42, 95% CI: 0.36–0.48, *P* < .001). Additionally, compared to other groups, hypertensive patients with short-term sitting (≤6 hours/day) and insufficiently active PA (0 < PA < 600 MET-minute/week) had the lowest cardiovascular mortality risk (HR = 0.36, 95% CI: 0.24–0.53, *P* < .001). The Kaplan–Meier curves (Fig. 2) indicated that hypertensive patients with short-term sitting (≤6 hours/day) and active PA (PA ≥ 600 MET-minute/week) demonstrated significantly higher overall survival probabilities. In contrast, those with long-term sitting (>6 hours/day) and inactive PA (PA = 0 MET-minute/week) showed the lowest overall and cardiovascular survival probabilities (Log-rank *P* < .001). In the sensitivity analysis, the association remained robust after excluding participants who died within 24 months (Table S3, Supplemental Digital Content, https://links.lww.com/MD/R179).

**Table 3:** Joint association of PA and SB with all-cause, and cardiovascular mortality among individuals with hypertension.

Characteristic	Death/no.	Model 1		Model 2		Model 3	
		95% CI	*P*	95% CI	*P*	95% CI	*P*
PA and SB							
All-cause mortality							
LS-INA	781/2746	–		–		–	
LS-AC	241/2980	0.22 (0.18–0.26)	<.001	0.34 (0.29–0.40)	<.001	0.43 (0.36–0.51)	<.001
LS-IA	154/1147	0.42 (0.32–0.55)	<.001	0.59 (0.47–0.76)	<.001	0.7 (0.55–0.89)	.003
SS-AC	635/6760	0.25 (0.22–0.29)	<.001	0.35 (0.30–0.40)	<.001	0.42 (0.36–0.48)	<.001
SS-IA	210/1494	0.35 (0.29–0.42)	<.001	0.39 (0.33–0.46)	<.001	0.45 (0.38–0.53)	<.001
SS-INA	599/3152	0.63 (0.55–0.72)	<.001	0.62 (0.55–0.71)	<.001	0.65 (0.57–0.75)	<.001
Cardiovascular mortality							
LS-INA	218/2746	–		–		–	
LS-AC	65/2980	0.21 (0.15–0.30)	<.001	0.36 (0.25–0.52)	<.001	0.48 (0.33–0.69)	<.001
LS-IA	33/1147	0.3 (0.19–0.49)	<.001	0.47 (0.29–0.74)	.001	0.58 (0.37–0.90)	.014
SS-AC	167/6760	0.23 (0.17–0.31)	<.001	0.33 (0.25–0.44)	<.001	0.43 (0.33–0.57)	<.001
SS-IA	42/1494	0.25 (0.18–0.36)	<.001	0.29 (0.20–0.42)	<.001	0.36 (0.24–0.53)	<.001
SS-INA	143/3152	0.53 (0.41–0.68)	<.001	0.52 (0.41–0.66)	<.001	0.57(0.44, 0.73)	<.001

Model 1 served as the unadjusted analysis; Model 2: adjusted for age, sex, and race; Model 3: fully adjusted model, including additional covariates such as marital status, education attainment, family income, alcohol intake, smoking status, BMI, sitting time, diabetes, and CVD history (congestive heart failure, coronary heart disease, angina pectoris, and stroke). *P*-value <.05 was considered significant.

BMI = body mass index; CI = confidence interval; CVD = cardiovascular disease; HR = hazard ratio; LS-AC = long-term sitting, >6 hours/day and active, PA ≥ 600 MET-minute/week; LS-IA = long-term sitting, >6 hours/day and insufficiently active, 0 < PA < 600 MET-minute/week; LS-INA = long-term sitting, >6 hours/day and inactive, PA = 0; PA = physical activity; SB = sedentary behavior; SS-AC = short-term sitting, ≤6 hours/day and active, PA ≥ 600 MET-minute/week; SS-IA = short-term sitting, ≤6 hours/day and insufficiently active, 0 < PA < 600 MET-minute/week; SS-INA = short-term sitting, ≤6 hours/day and inactive, PA = 0.

### 3.4. Subgroup analysis

In the subgroup analysis by sex, the joint effects of PA and SB on mortality rates did not show significant differences between the gender groups (Table S4, Supplemental Digital Content, https://links.lww.com/MD/R179). In the age subgroup analysis, the combined impact of short-term sitting (≤6 hours/day) and active PA (PA ≥ 600 MET-minute/week) was found to be more beneficial for hypertensive individuals aged 60 years and older compared to those younger than 60 (Table S5, Supplemental Digital Content, https://links.lww.com/MD/R179).

## 4. Discussion

Hypertension, often referred to as the “silent killer” due to its insidious onset and severe consequences, carries a lifetime risk exceeding 80% and is a major risk factor for both all-cause and cardiovascular mortality.^[6]^ Global data indicate that over one billion individuals are affected by hypertension, and these individuals face a substantially higher risk of cardiovascular events compared to non-hypertensive populations.^[33]^ Despite advancements in modern medicine and increasingly sophisticated treatment and management strategies for hypertension, the rates of all-cause and cardiovascular mortality remain high among hypertensive patients. This underscores the urgent need to explore novel lifestyle interventions aimed at improving survival outcomes in this population.

In this study, we conducted a preliminary investigation into the combined effects of SB and PA on mortality risk among hypertensive individuals. We found that approximately 53.3% of these hypertensive individuals engaged in sufficient physical activity (PA ≥ 600 MET-minute/week), while 26.6% reported sitting for <4 hours/day. Over an average follow-up period of 6.17 years, active PA was associated with a reduced risk of all-cause and cardiovascular mortality, whereas sitting for <4 hours/day was also linked to lower risks for both types of mortality. In the combined analysis, hypertensive individuals who were inactive (PA = 0 MET-minute/week) and had sedentary time exceeding 6 hours/day exhibited a higher risk of all-cause and cardiovascular mortality compared to those with only one risk factor. This association remained significant in sensitivity and subgroup analyses. To our knowledge, this is the first study to examine the combined effects of SB and PA on mortality risk among hypertensive individuals in the United States. Our findings emphasized the importance of considering both SB and PA in the follow-up management of hypertensive populations.

Previous research by Ge et al^[21]^ observed that moderate-intensity aerobic exercise is associated with a lower risk of all-cause mortality in hypertensive patients, with more pronounced benefits seen in those with longer sedentary durations. Notably, our study provided additional evidence supporting the protective role of PA in hypertensive individuals, regardless of the type of activity. In contrast, we identified that active PA is significantly associated with a reduced risk of cardiovascular mortality specifically related to hypertension, which partially diverges from Ge findings. Moreover, we found that hypertensive patients engaging in more than 600 MET-min of PA per week demonstrated better survival probabilities compared to their inactive counterparts. Among various subgroups, hypertensive patients with a weekly PA of 2000 to 4000 MET-min exhibited the lowest all-cause mortality risk and minimal cardiovascular mortality risk. These findings align with a previous study focusing on the general population.^[34]^ Thus, our results suggested that maintaining moderate-intensity PA can yield superior benefits in reducing mortality risk among hypertensive patients.

Additionally, the association between SB and mortality has been explored within hypertensive populations.^[19,35]^ Our data indicated that sitting for <4 hours/day may be associated with a reduction in risks for both all-cause and cardiovascular mortality, consistent with some prior findings. The combined analysis allowed us to explore the unique and collective contributions of each factor to mortality outcomes, providing comprehensive survival guidance for hypertensive patients. Specifically, our findings indicated that increasing PA while reducing SB can significantly lower the risks of all-cause and cardiovascular mortality in this population compared to lifestyle interventions focused solely on one aspect.

Several potential biological pathways may explain our findings. In hypertensive populations, low PA exacerbates cardiovascular risk by disrupting several vital physiological processes that regulate BP and vascular health. For instance, insufficient PA is linked to compromised endothelial function, as it reduces nitric oxide bioavailability (a molecule essential for vasodilation and maintaining vascular tone). Diminished endothelial performance escalates vascular resistance, directly contributing to elevated BP.^[36,37]^ Furthermore, physical inactivity can lead to heightened sympathetic nervous system activity, which increases heart rate and induces vasoconstriction, establishing a cycle of sustained hypertension.^[38,39]^ Moreover, limited PA is associated with activation of the renin–angiotensin–aldosterone system, intensifying vasoconstriction and promoting sodium and fluid retention, both of which are critical factors in the development and persistence of hypertension.^[40,41]^ Additionally, prolonged physical inactivity exacerbates metabolic dysfunctions, such as insulin resistance and dyslipidemia, which are prevalent among patients with hypertension and significantly heighten cardiovascular risk. Evidence indicates that regular PA can mitigate inflammatory responses by reducing levels of C-reactive protein and interleukin-6, thereby contributing to cardiovascular risk reduction. However, research indicates that SB significantly impacts metabolic health, leading to decreased lipoprotein lipase activity, elevated serum triglyceride levels, and increased low-density lipoprotein levels, all of which elevate cardiovascular risk.^[42,43]^ Additionally, prolonged SB is associated with decreased insulin sensitivity, a key factor contributing to increased mortality risk. It may also lead to venous pooling, resulting in increased vascular resistance and altered BP, thereby further exacerbating cardiovascular risk.^[44–46]^

Overall, the interaction between PA and SB underscores the importance of a dual intervention approach. Research suggests that while SB has detrimental health effects, high levels of PA (particularly 60–75 minutes of moderate to vigorous exercise per day) can partially counteract these risks. For example, a large prospective study found that adequate PA reduces the mortality risk associated with SB, especially for cardiovascular disease and diabetes-related mortality.^[47]^ Other studies have shown that even with regular vigorous PA, prolonged SB (such as >8 hours) still poses health risks. Regularly interrupting SB has been shown to significantly enhance BP control, metabolic health,^[48,49]^ and cognitive function,^[50]^ while also reducing overall mortality risk. Engaging in brief activities, such as standing or moving for a few minutes every 30 minutes, can effectively improve health outcomes.

Despite the valuable insights provided, this study is subject to certain limitations. Firstly, the assessment of PA and SB relied on self-reported data collected at a single time point. This approach is susceptible to measurement bias and does not capture behavioral changes over time. Secondly, information on antihypertensive medication was limited to self-reported use and lacked details on specific drug classes and dosages. This may represent an unmeasured confounding factor. Furthermore, the analysis could not account for variations in PA intensity due to data constraints. Future research incorporating objective, repeated behavioral measures and detailed pharmacological data would help validate and extend these findings.

## 5. Conclusions

This study demonstrated that the combination of active PA and reduced SB is significantly associated with lower risks of all-cause and cardiovascular mortality among individuals with hypertension. A comprehensive evaluation of both PA and SB may provide deeper insights into the impact of lifestyle interventions on survival outcomes in this population.

## Author contributions

**Conceptualization:** Wenjing Zhang.

**Data curation:** Zizhen Chen, Mengxue Wang.

**Formal analysis:** Yihang Du, Zizhen Chen, Mengxue Wang.

**Funding acquisition:** Yuanhui Hu.

**Methodology:** Wenjing Zhang, Yihang Du, Ting Zhao, Yuanhui Hu.

**Supervision:** Ting Zhao, Yuanhui Hu.

**Validation:** Ting Zhao, Yuanhui Hu.

**Visualization:** Wenjing Zhang.

**Writing – original draft:** Wenjing Zhang, Yihang Du, Zizhen Chen, Mengxue Wang.

**Writing – review & editing:** Ting Zhao, Yuanhui Hu.

## Supplementary Material



## References

[1] Gao G Chen Z Yan G Bao M. Impact of hypertensive heart disease, risk factors, and age-period-cohort models across 204 nations and regions from 1990 to 2019: a global perspective from the 2019 global burden of disease study. Front Cardiovasc Med. 2024;11:1417523. 39091356 10.3389/fcvm.2024.1417523 PMC11291211

[2] Martin SS Aday AW Almarzooq ZI. 2024 Heart disease and stroke statistics: a report of us and global data from the American Heart Association. Circulation. 2024;149:e347–913. 38264914 10.1161/CIR.0000000000001209 PMC12146881

[3] He J Zhu Z Bundy JD Dorans KS Chen J Hamm LL. Trends in cardiovascular risk factors in US adults by race and ethnicity and socioeconomic status, 1999–2018. JAMA. 2021;326:1286–98. 34609450 10.1001/jama.2021.15187 PMC8493438

[4] Chen Y Li Y Liu M Xu W Tong S Liu K. Association between systemic immunity-inflammation index and hypertension in US adults from NHANES 1999–2018. Sci Rep. 2024;14:5677. 38454104 10.1038/s41598-024-56387-6 PMC10920861

[5] Virani SS Alonso A Aparicio HJ. Heart disease and stroke statistics-2021 update: a report from the American Heart Association. Circulation. 2021;143:e254–743. 33501848 10.1161/CIR.0000000000000950 PMC13036842

[6] Whelton PK Carey RM Aronow WS. 2017 ACC/AHA/AAPA/ABC/ACPM/AGS/APhA/ASH/ASPC/NMA/PCNA guideline for the prevention, detection, evaluation, and management of high blood pressure in adults: a report of the American College of Cardiology/American Heart Association task force on clinical practice guidelines. J Am Coll Cardiol. 2018;71:e127–248. 29146535 10.1016/j.jacc.2017.11.006

[7] Mills KT Bundy JD Kelly TN. Global disparities of hypertension prevalence and control: a systematic analysis of population-based studies from 90 countries. Circulation. 2016;134:441–50. 27502908 10.1161/CIRCULATIONAHA.115.018912 PMC4979614

[8] Wang H Zhang Y Sun M Chen Z. Nonlinear association of physical activity with early cardiovascular–kidney–metabolic syndrome risk: a comprehensive analysis of NHANES data from 2007 to 2018. BMC Public Health. 2025;25:1965. 40437440 10.1186/s12889-025-23157-6 PMC12117810

[9] Haskell WL Lee IM Pate RR. Physical activity and public health: updated recommendation for adults from the American College of Sports Medicine and the American Heart Association. Circulation. 2007;116:1081–93. 17671237 10.1161/CIRCULATIONAHA.107.185649

[10] Cornelissen VA Smart NA. Exercise training for blood pressure: a systematic review and meta-analysis. J Am Heart Assoc. 2013;2:e004473. 23525435 10.1161/JAHA.112.004473 PMC3603230

[11] Strath SJ Kaminsky LA Ainsworth BE. Guide to the assessment of physical activity: clinical and research applications: a scientific statement from the American Heart Association. Circulation. 2013;128:2259–79. 24126387 10.1161/01.cir.0000435708.67487.da

[12] Barone Gibbs B Hivert MF Jerome GJ. Physical activity as a critical component of first-line treatment for elevated blood pressure or cholesterol: who, what, and how?: a scientific statement from the American Heart Association. Hypertension. 2021;78:e26–37. 34074137 10.1161/HYP.0000000000000196

[13] Kraus WE Bittner V Appel L. The national physical activity plan: a call to action from the American Heart Association: a science advisory from the American Heart Association. Circulation. 2015;131:1932–40. 25918126 10.1161/CIR.0000000000000203

[14] Bull FC Al-Ansari SS Biddle S. World Health Organization 2020 guidelines on physical activity and sedentary behaviour. Br J Sports Med. 2020;54:1451–62. 33239350 10.1136/bjsports-2020-102955 PMC7719906

[15] Owen N Healy GN Dempsey PC. Sedentary behavior and public health: integrating the evidence and identifying potential solutions. Annu Rev Public Health. 2020;41:265–87. 31913771 10.1146/annurev-publhealth-040119-094201

[16] Brakenridge CJ Koster A de Galan BE. Associations of 24 h time-use compositions of sitting, standing, physical activity and sleeping with optimal cardiometabolic risk and glycaemic control: the Maastricht Study. Diabetologia. 2024;67:1356–67. 38656371 10.1007/s00125-024-06145-0 PMC11153304

[17] Katzmarzyk PT Powell KE Jakicic JM. Sedentary behavior and health: update from the 2018 physical activity guidelines advisory committee. Med Sci Sports Exerc. 2019;51:1227–41. 31095080 10.1249/MSS.0000000000001935 PMC6527341

[18] Madden KM Feldman B Chase J. Sedentary time and metabolic risk in extremely active older adults. Diabetes Care. 2021;44:194–200. 33067259 10.2337/dc20-0849 PMC7783925

[19] Boudreaux BD Romero EK Diaz KM. Sedentary behavior and risk of cardiovascular disease and all-cause mortality in United States adults with hypertension. J Hypertens. 2023;41:1793–801. 37605821 10.1097/HJH.0000000000003540

[20] Sha S Bu XP Wang AW Chen HZ. Association between inflammatory biomarkers and hypertension among sedentary adults in US: NHANES 2009–2018. J Clin Hypertens (Greenwich). 2024;26:945–54. 38946147 10.1111/jch.14851 PMC11301436

[21] Ge C Long B Lu Q Jiang Z He Y. Associations of different type of physical activity with all-cause mortality in hypertension participants. Sci Rep. 2024;14:7515. 38553535 10.1038/s41598-024-58197-2 PMC10980699

[22] Dempsey PC Larsen RN Dunstan DW Owen N Kingwell BA. Sitting less and moving more: implications for hypertension. Hypertension. 2018;72:1037–46. 30354827 10.1161/HYPERTENSIONAHA.118.11190 PMC7343526

[23] Yang L Cao C Kantor ED. Trends in sedentary behavior among the US population, 2001–2016. JAMA. 2019;321:1587–97. 31012934 10.1001/jama.2019.3636 PMC6487546

[24] Saunders TJ McIsaac T Douillette K. Sedentary behaviour and health in adults: an overview of systematic reviews. Appl Physiol Nutr Metab. 2020;45(Suppl. 2):S197–217. 33054341 10.1139/apnm-2020-0272

[25] Riquelme R Rezende LFM Marques A Drenowatz C Ferrari G. Association between 24-h movement guidelines and cardiometabolic health in Chilean adults. Sci Rep. 2022;12:5805. 35388103 10.1038/s41598-022-09729-1 PMC8986846

[26] Piercy KL Troiano RP Ballard RM. The physical activity guidelines for Americans. JAMA. 2018;320:2020–8. 30418471 10.1001/jama.2018.14854 PMC9582631

[27] An X Li J Li Y. Combined influence of physical activity and C-reactive protein to albumin ratio on mortality among older cancer survivors in the United States: a prospective cohort study. Eur Rev Aging Phys Act. 2024;21:26. 39358685 10.1186/s11556-024-00361-8 PMC11448037

[28] Wei X Min Y Xiang Z Zeng Y Wang J Liu L. Joint association of physical activity and dietary quality with survival among US cancer survivors: a population-based cohort study. Int J Surg. 2024;110:5585–94. 38874488 10.1097/JS9.0000000000001636 PMC11392114

[29] Yu C Cao Y Liu Q. Sitting time, leisure-time physical activity, and risk of mortality among US stroke survivors: a prospective cohort study from the NHANES 2007 to 2018. Stroke. 2025;56:1738–47. 40321136 10.1161/STROKEAHA.124.049672

[30] Dai W Albrecht SS. Sitting time and its interaction with physical activity in relation to all-cause and heart disease mortality in US adults with diabetes. Diabetes Care. 2024;47:1764–8. 39028423 10.2337/dc24-0673 PMC11417277

[31] Whelton PK Carey RM Aronow WS. 2017 ACC/AHA/AAPA/ABC/ACPM/AGS/APhA/ASH/ASPC/NMA/PCNA guideline for the prevention, detection, evaluation, and management of high blood pressure in adults: a report of the American College of Cardiology/American Heart Association task force on clinical practice guidelines. Hypertension. 2018;71:e13–e115. 29133356 10.1161/HYP.0000000000000065

[32] Global guideline for type 2 diabetes. Diabetes research and clinical practice. Diabetes Res Clin Pract. 2014;104:1–52. 24508150 10.1016/j.diabres.2012.10.001

[33] NCD Risk Factor Collaboration (NCD-RisC). Worldwide trends in blood pressure from 1975 to 2015: a pooled analysis of 1479 population-based measurement studies with 19·1 million participants. Lancet. 2017;389:37–55. 27863813 10.1016/S0140-6736(16)31919-5 PMC5220163

[34] Arem H Moore SC Patel A. Leisure time physical activity and mortality: a detailed pooled analysis of the dose–response relationship. JAMA Intern Med. 2015;175:959–67. 25844730 10.1001/jamainternmed.2015.0533 PMC4451435

[35] Wang H Zhou Z Zhang L. Sedentary behavior modified the association between depression and risk of all-cause deaths in hypertensive population. J Hypertens. 2025;43:474–80. 39887980 10.1097/HJH.0000000000003929

[36] Green DJ Hopman MT Padilla J Laughlin MH Thijssen DH. Vascular adaptation to exercise in humans: role of hemodynamic stimuli. Physiol Rev. 2017;97:495–528. 28151424 10.1152/physrev.00014.2016 PMC5539408

[37] da Silva RSN da Silva DS Waclawovsky G Schaun MI. Effects of aerobic, resistance, and combined training on endothelial function and arterial stiffness in older adults: study protocol for a systematic review and meta-analysis. Syst Rev. 2022;11:171. 35964075 10.1186/s13643-022-02036-w PMC9375352

[38] Fagard RH. Exercise therapy in hypertensive cardiovascular disease. Prog Cardiovasc Dis. 2011;53:404–11. 21545926 10.1016/j.pcad.2011.03.006

[39] Diaz KM Shimbo D. Physical activity and the prevention of hypertension. Curr Hypertens Rep. 2013;15:659–68. 24052212 10.1007/s11906-013-0386-8 PMC3901083

[40] Kaschina E. Cross-talk between exercises and renin–angiotensin–aldosterone-system blockade in hypertension. Hypertens Res. 2024;47:1981–3. 38750223 10.1038/s41440-024-01688-6 PMC11224010

[41] Baffour-Awuah B Man M Goessler KF. Effect of exercise training on the renin–angiotensin–aldosterone system: a meta-analysis. J Hum Hypertens. 2024;38:89–101. 38017087 10.1038/s41371-023-00872-4 PMC10844078

[42] Crichton GE Alkerwi A. Physical activity, sedentary behavior time and lipid levels in the observation of cardiovascular risk factors in Luxembourg study. Lipids Health Dis. 2015;14:87. 26256803 10.1186/s12944-015-0085-3 PMC4530482

[43] Higgins S Pomeroy A Bates LC. Sedentary behavior and cardiovascular disease risk: an evolutionary perspective. Front Physiol. 2022;13:962791. 35965885 10.3389/fphys.2022.962791 PMC9363656

[44] Hoffmann SW Schierbauer J Zimmermann P. Effects of interrupting prolonged sitting with light-intensity physical activity on inflammatory and cardiometabolic risk markers in young adults with overweight and obesity: secondary outcome analyses of the SED-ACT randomized controlled crossover trial. Biomolecules. 2024;14:1029–e18. 39199416 10.3390/biom14081029 PMC11352707

[45] Andersson DP Kerr AG Dahlman I Rydén M Arner P. Relationship between a sedentary lifestyle and adipose insulin resistance. Diabetes. 2023;72:316–25. 36445942 10.2337/db22-0612

[46] Jia H Liu Y Liu D. Role of leisure sedentary behavior on type 2 diabetes and glycemic homeostasis: a Mendelian randomization analysis. Front Endocrinol (Lausanne). 2023;14:1221228. 38075044 10.3389/fendo.2023.1221228 PMC10702218

[47] Ekelund U Steene-Johannessen J Brown WJ. Does physical activity attenuate, or even eliminate, the detrimental association of sitting time with mortality? A harmonised meta-analysis of data from more than 1 million men and women. Lancet. 2016;388:1302–10. 27475271 10.1016/S0140-6736(16)30370-1

[48] Baddou I El Hamdouchi A El Harchaoui I. Objectively measured physical activity and sedentary time among children and adolescents in morocco: a cross-sectional study. Biomed Res Int. 2018;2018:8949757. 30356414 10.1155/2018/8949757 PMC6178184

[49] Gale JT Wei DL Haszard JJ Brown RC Taylor RW Peddie MC. Breaking up evening sitting with resistance activity improves postprandial glycemic response: a randomized crossover study. Med Sci Sports Exerc. 2023;55:1471–80. 36921112 10.1249/MSS.0000000000003166 PMC10348652

[50] Chauntry AJ Bishop NC Hamer M Paine NJ. Frequently interrupting prolonged sitting with light body-weighted resistance activity alters psychobiological responses to acute psychological stress: a randomized crossover trial. Ann Behav Med. 2023;57:301–12. 36005837 10.1093/abm/kaac055

