# Supplementary material for: Combined associations of physical activity and sedentary behavior with all-cause and cardiovascular mortality in hypertensive adults: A cohort study using NHANES data (2007–2018)
Source: Medicine (Baltimore). 2026 Jan 23;105(4):e47167. doi: 10.1097/MD.0000000000047167 (PMC12851722; doi:10.1097/MD.0000000000047167)
Supplement: Supplementary file 1 [file medi-105-e47167-s001.docx]

**Supplementary Table 1** Baseline Characteristics of NHANES individuals with hypertension by SB levels.

| **Characteristic** | **Overall,** (n=18279) Weighted%:100.0 | **SB levels** | | | ***P*** |
| --- | --- | --- | --- | --- | --- |
|  |  | **>8h/day**, (n=3606) Weighted%:19.7 | **>=4h/day& <=8h/day**, (n=9808) Weighted%:53.7 | **<4h/day**, (n=4865) Weighted%:26.6 |  |
| **Sex** |  |  |  |  | 0.3 |
| *Female* | 8,798 (48%) | 1,710 (47%) | 4,728 (49%) | 2,360 (47%) |  |
| *Male* | 9,481 (52%) | 1,896 (53%) | 5,080 (51%) | 2,505 (53%) |  |
| **Age(year)** | 55 (43, 67) | 54 (43, 65) | 57 (45, 68) | 53 (41, 64) | **<0.001** |
| **Age group** |  |  |  |  | **<0.001** |
| *<60 years* | 9,186 (60%) | 1,930 (63%) | 4,659 (56%) | 2,597 (64%) |  |
| *60-69 years* | 4,023 (18%) | 719 (17%) | 2,143 (19%) | 1,161 (18%) |  |
| *70-79 years* | 2,715 (12%) | 427 (9.3%) | 1,638 (14%) | 650 (10%) |  |
| *80+ years* | 2,355 (10.0%) | 530 (10%) | 1,368 (11%) | 457 (7.3%) |  |
| **Race** |  |  |  |  | **<0.001** |
| *Mexican American* | 2,395 (6.9%) | 253 (3.6%) | 1,107 (5.9%) | 1,035 (13%) |  |
| *Other Hispanic* | 1,779 (4.9%) | 222 (2.8%) | 870 (4.6%) | 687 (8.1%) |  |
| *Non-Hispanic White* | 7,560 (68%) | 1,777 (75%) | 4,273 (69%) | 1,510 (56%) |  |
| *Non-Hispanic Black* | 4,570 (13%) | 939 (12%) | 2,487 (13%) | 1,144 (15%) |  |
| *Other/multiracial* | 1,975 (7.6%) | 415 (7.0%) | 1,071 (7.5%) | 489 (8.3%) |  |
| **Marital status** |  |  |  |  | 0.2 |
| *Married* | 9,641 (57%) | 1,792 (56%) | 5,227 (58%) | 2,622 (57%) |  |
| *Never married* | 2,269 (12%) | 493 (13%) | 1,189 (12%) | 587 (12%) |  |
| *Living with partner* | 1,113 (6.4%) | 209 (6.0%) | 567 (6.2%) | 337 (7.1%) |  |
| *Other* | 5,256 (24%) | 1,112 (25%) | 2,825 (24%) | 1,319 (24%) |  |
| **Education attainment** |  |  |  |  | **<0.001** |
| *High school or less* | 9,356 (42%) | 1,389 (30%) | 4,947 (43%) | 3,020 (54%) |  |
| *Some College* | 5,231 (32%) | 1,152 (34%) | 2,856 (31%) | 1,223 (30%) |  |
| *College Graduate or above* | 3,692 (26%) | 1,065 (36%) | 2,005 (26%) | 622 (16%) |  |
| **Family income** |  |  |  |  | **<0.001** |
| *high income* | 3,216 (27%) | 886 (35%) | 1,734 (26%) | 596 (19%) |  |
| *low income* | 6,545 (25%) | 1,050 (19%) | 3,449 (24%) | 2,046 (32%) |  |
| *medium income* | 8,518 (48%) | 1,670 (46%) | 4,625 (49%) | 2,223 (49%) |  |
| **Alq group** |  |  |  |  | **<0.001** |
| *Non-drinker* | 5,647 (24%) | 968 (21%) | 2,965 (24%) | 1,714 (28%) |  |
| *1-5 drinks/month* | 8,706 (48%) | 1,793 (49%) | 4,723 (49%) | 2,190 (46%) |  |
| *5-10 drinks/month* | 1,235 (8.3%) | 265 (9.0%) | 643 (7.9%) | 327 (8.4%) |  |
| *10+ drinks/month* | 2,691 (19%) | 580 (21%) | 1,477 (19%) | 634 (18%) |  |
| **Smoke group** |  |  |  |  | **<0.001** |
| *Never smoker* | 9,537 (52%) | 1,825 (53%) | 5,023 (52%) | 2,689 (52%) |  |
| *Former smoker* | 5,213 (29%) | 1,111 (31%) | 2,874 (30%) | 1,228 (27%) |  |
| *Current smoker* | 3,529 (19%) | 670 (17%) | 1,911 (19%) | 948 (21%) |  |
| **BMI** | 29 (26, 34) | 31 (27, 36) | 29 (26, 34) | 29 (25, 33) | **<0.001** |
| **BMI group** |  |  |  |  | **<0.001** |
| *Normal(18.5 to <25)* | 3,754 (20%) | 643 (17%) | 1,996 (20%) | 1,115 (24%) |  |
| *Obese(30 or greater)* | 8,371 (47%) | 1,885 (53%) | 4,495 (46%) | 1,991 (41%) |  |
| *Overweight(25 to <30)* | 5,958 (33%) | 1,036 (30%) | 3,212 (33%) | 1,710 (34%) |  |
| *Underweight(<18.5)* | 196 (1.0%) | 42 (0.8%) | 105 (1.0%) | 49 (1.1%) |  |
| **PA** | 960 (0, 3,720) | 360 (0, 1,620) | 1,120 (0, 3,800) | 2,160 (240, 7,680) | **<0.001** |
| **PA group** |  |  |  |  | **<0.001** |
| *Inactive* | 5,898 (28%) | 1,552 (39%) | 3,077 (26%) | 1,269 (21%) |  |
| *Insufficiently active* | 2,641 (14%) | 639 (18%) | 1,395 (14%) | 607 (11%) |  |
| *Active* | 9,740 (58%) | 1,415 (43%) | 5,336 (60%) | 2,989 (68%) |  |
| **Diabetes** |  |  |  |  | **<0.001** |
| *Yes* | 4,828 (21%) | 1,011 (23%) | 2,593 (21%) | 1,224 (18%) |  |
| *No* | 13,451 (79%) | 2,595 (77%) | 7,215 (79%) | 3,641 (82%) |  |
| **Congestive heart failure** |  |  |  |  | **<0.001** |
| *Yes* | 991 (4.2%) | 270 (5.6%) | 552 (4.2%) | 169 (2.9%) |  |
| *No* | 17,288 (96%) | 3,336 (94%) | 9,256 (96%) | 4,696 (97%) |  |
| **Coronary heart disease** |  |  |  |  | **0.013** |
| *Yes* | 1,172 (5.6%) | 272 (6.0%) | 668 (6.0%) | 232 (4.4%) |  |
| *No* | 17,107 (94%) | 3,334 (94%) | 9,140 (94%) | 4,633 (96%) |  |
| **Angina pectoris** |  |  |  |  | 0.7 |
| *Yes* | 713 (3.6%) | 163 (3.9%) | 402 (3.6%) | 148 (3.3%) |  |
| *No* | 17,566 (96%) | 3,443 (96%) | 9,406 (96%) | 4,717 (97%) |  |
| **Stroke** |  |  |  |  | **0.019** |
| *Yes* | 1,124 (4.9%) | 279 (5.6%) | 636 (5.0%) | 209 (3.8%) |  |
| *No* | 17,155 (95%) | 3,327 (94%) | 9,172 (95%) | 4,656 (96%) |  |
| **Cancer** |  |  |  |  | **<0.001** |
| *Yes* | 2,377 (14%) | 518 (15%) | 1,412 (15%) | 447 (10%) |  |
| *No* | 15,902 (86%) | 3,088 (85%) | 8,396 (85%) | 4,418 (90%) |  |
| **All-cause mortality** | 2,620 (11%) | 628 (13%) | 1,470 (11%) | 522 (8.4%) | **<0.001** |
| **Cardiovascular mortality** | 668 (2.7%) | 156 (3.1%) | 406 (3.0%) | 106 (1.6%) | **<0.001** |
| Abbreviations: BMI: Body Mass Index; SB: Sedentary Behavior; PA: Physical Activity；Median (Q1, Q3); n (%); *P <0.05 was considered significant. | | | | | |

**Supplementary Table 2** Baseline Characteristics of NHANES individuals with hypertension by PA levels.

| **Characteristic** | **Overall,** (n=18279) Weighted | **PA levels** | | | ***P*** |
| --- | --- | --- | --- | --- | --- |
|  |  | **Inactive,** (n=5898) Weighted%:32.3 | **Insufficiently active,** (n=2641) Weighted%:14.4 | **Active,** (n=9740) Weighted%:53.3 |  |
| **Sex** |  |  |  |  | **<0.001** |
| *Female* | 8,798 (48%) | 3,412 (60%) | 1,471 (56%) | 3,915 (40%) |  |
| *Male* | 9,481 (52%) | 2,486 (40%) | 1,170 (44%) | 5,825 (60%) |  |
| **Age(year)** | 55 (43, 67) | 61 (50, 73) | 56 (45, 68) | 53 (40, 63) | **<0.001** |
| **Age group** |  |  |  |  | **<0.001** |
| *<60 years* | 9,186 (60%) | 2,169 (46%) | 1,263 (57%) | 5,754 (67%) |  |
| *60-69 years* | 4,023 (18%) | 1,411 (20%) | 615 (19%) | 1,997 (17%) |  |
| *70-79 years* | 2,715 (12%) | 1,101 (16%) | 438 (14%) | 1,176 (9.6%) |  |
| *80+ years* | 2,355 (10.0%) | 1,217 (18%) | 325 (10%) | 813 (6.1%) |  |
| **Race** |  |  |  |  | **<0.001** |
| *Mexican American* | 2,395 (6.9%) | 867 (7.7%) | 309 (5.9%) | 1,219 (6.8%) |  |
| *Other Hispanic* | 1,779 (4.9%) | 617 (5.2%) | 240 (4.4%) | 922 (4.9%) |  |
| *Non-Hispanic White* | 7,560 (68%) | 2,373 (65%) | 1,039 (66%) | 4,148 (69%) |  |
| *Non-Hispanic Black* | 4,570 (13%) | 1,504 (14%) | 715 (14%) | 2,351 (12%) |  |
| *Other/multiracial* | 1,975 (7.6%) | 537 (7.5%) | 338 (9.2%) | 1,100 (7.2%) |  |
| **Marital status** |  |  |  |  | **<0.001** |
| *Married* | 9,641 (57%) | 2,946 (54%) | 1,400 (59%) | 5,295 (59%) |  |
| *Never married* | 2,269 (12%) | 566 (9.5%) | 312 (11%) | 1,391 (14%) |  |
| *Living with partner* | 1,113 (6.4%) | 269 (5.2%) | 129 (5.3%) | 715 (7.2%) |  |
| *Other* | 5,256 (24%) | 2,117 (31%) | 800 (25%) | 2,339 (20%) |  |
| **Education attainment** |  |  |  |  | **<0.001** |
| *High school or less* | 9,356 (42%) | 3,577 (52%) | 1,286 (38%) | 4,493 (39%) |  |
| *Some College* | 5,231 (32%) | 1,475 (29%) | 768 (32%) | 2,988 (33%) |  |
| *College Graduate or above* | 3,692 (26%) | 846 (19%) | 587 (30%) | 2,259 (29%) |  |
| **Family income** |  |  |  |  | **<0.001** |
| *high income* | 3,216 (27%) | 782 (21%) | 471 (28%) | 1,963 (30%) |  |
| *low income* | 6,545 (25%) | 2,385 (30%) | 975 (25%) | 3,185 (22%) |  |
| *medium income* | 8,518 (48%) | 2,731 (50%) | 1,195 (47%) | 4,592 (48%) |  |
| **Alq group** |  |  |  |  | **<0.001** |
| *Non-drinker* | 5,647 (24%) | 2,299 (34%) | 848 (24%) | 2,500 (20%) |  |
| *1-5 drinks/month* | 8,706 (48%) | 2,725 (48%) | 1,276 (48%) | 4,705 (48%) |  |
| *5-10 drinks/month* | 1,235 (8.3%) | 267 (5.4%) | 157 (8.1%) | 811 (9.7%) |  |
| *10+ drinks/month* | 2,691 (19%) | 607 (13%) | 360 (20%) | 1,724 (22%) |  |
| **Smoke group** |  |  |  |  | 0.8 |
| *Never smoker* | 9,537 (52%) | 3,061 (51%) | 1,406 (53%) | 5,070 (52%) |  |
| *Former smoker* | 5,213 (29%) | 1,721 (30%) | 767 (29%) | 2,725 (29%) |  |
| *Current smoker* | 3,529 (19%) | 1,116 (19%) | 468 (18%) | 1,945 (19%) |  |
| **BMI** | 29 (26, 34) | 30 (26, 36) | 30 (26, 35) | 29 (25, 33) | **<0.001** |
| **BMI group** |  |  |  |  | **<0.001** |
| *Normal(18.5 to <25)* | 3,754 (20%) | 1,100 (17%) | 542 (19%) | 2,112 (21%) |  |
| *Obese(30 or greater)* | 8,371 (47%) | 2,900 (53%) | 1,221 (47%) | 4,250 (44%) |  |
| *Overweight(25 to <30)* | 5,958 (33%) | 1,831 (29%) | 837 (32%) | 3,290 (34%) |  |
| *Underweight(<18.5)* | 196 (1.0%) | 67 (1.1%) | 41 (1.4%) | 88 (0.8%) |  |
| **SB** | 6 (4, 8) | 7 (4, 10) | 6 (4, 10) | 5 (3, 8) | **<0.001** |
| **SB group** |  |  |  |  | **<0.001** |
| *>8h/day* | 3,606 (23%) | 1,552 (32%) | 639 (30%) | 1,415 (17%) |  |
| *>=4h/day& <=8h/day* | 9,808 (55%) | 3,077 (51%) | 1,395 (53%) | 5,336 (57%) |  |
| *<4h/day* | 4,865 (22%) | 1,269 (17%) | 607 (18%) | 2,989 (26%) |  |
| **Diabetes** |  |  |  |  | **<0.001** |
| Yes | 4,828 (21%) | 2,059 (30%) | 749 (22%) | 2,020 (16%) |  |
| No | 13,451 (79%) | 3,839 (70%) | 1,892 (78%) | 7,720 (84%) |  |
| **Congestive heart failure** |  |  |  |  | **<0.001** |
| Yes | 991 (4.2%) | 525 (7.6%) | 128 (4.0%) | 338 (2.7%) |  |
| No | 17,288 (96%) | 5,373 (92%) | 2,513 (96%) | 9,402 (97%) |  |
| **Coronary heart disease** |  |  |  |  | **<0.001** |
| Yes | 1,172 (5.6%) | 524 (8.2%) | 144 (4.7%) | 504 (4.6%) |  |
| No | 17,107 (94%) | 5,374 (92%) | 2,497 (95%) | 9,236 (95%) |  |
| **Angina pectoris** |  |  |  |  | **<0.001** |
| Yes | 713 (3.6%) | 315 (5.5%) | 88 (2.5%) | 310 (3.0%) |  |
| No | 17,566 (96%) | 5,583 (94%) | 2,553 (98%) | 9,430 (97%) |  |
| **Stroke** |  |  |  |  | **<0.001** |
| Yes | 1,124 (4.9%) | 584 (8.7%) | 149 (4.3%) | 391 (3.2%) |  |
| No | 17,155 (95%) | 5,314 (91%) | 2,492 (96%) | 9,349 (97%) |  |
| **Cancer** |  |  |  |  | **<0.001** |
| Yes | 2,377 (14%) | 919 (17%) | 338 (15%) | 1,120 (13%) |  |
| No | 15,902 (86%) | 4,979 (83%) | 2,303 (85%) | 8,620 (87%) |  |
| **All-cause mortality** | 2,620 (11%) | 1,380 (20%) | 364 (10%) | 876 (6.6%) | **<0.001** |
| **Cardiovascular mortality** | 668 (2.7%) | 361 (5.3%) | 75 (2.0%) | 232 (1.7%) | **<0.001** |
| Abbreviations: BMI: Body Mass Index; SB: Sedentary Behavior; PA: Physical Activity. Median (Q1, Q3); n (%); *P <0.05 was considered significant. | | | | | |

**Supplementary Table 3** Joint association of PA and SB with all-cause, and cardiovascular mortality among individuals with hypertension (Excluding the participants who died within 24 months).

| **Characteristic** | **Death/No.** | **Model 1** | | **Model 2** | | **Model 3** | |
| --- | --- | --- | --- | --- | --- | --- | --- |
|  |  | **95% CI** | ***p*** | **95% CI** | ***p*** | **95% CI** | ***p*** |
| **PA&SB** |  |  |  |  |  |  |  |
| **All-cause mortality** | | | | |  | |  |
| LS - INA | 565/2530 | — |  | — |  | — |  |
| LS - AC | 188/2927 | 0.24(0.19, 0.29) | **<0.001** | 0.37(0.30, 0.46) | **<0.001** | 0.47(0.38, 0.58) | **<0.001** |
| LS - IA | 119/1112 | 0.42(0.32, 0.54) | **<0.001** | 0.59(0.47, 0.75) | **<0.001** | 0.7(0.55, 0.88) | **0.003** |
| SS - AC | 516/6641 | 0.28(0.24, 0.32) | **<0.001** | 0.37(0.32, 0.43) | **<0.001** | 0.44(0.39, 0.51) | **<0.001** |
| SS - IA | 179/1463 | 0.39(0.32, 0.48) | **<0.001** | 0.43(0.36, 0.52) | **<0.001** | 0.49(0.40, 0.59) | **<0.001** |
| SS - INA | 466/3019 | 0.65(0.55, 0.77) | **<0.001** | 0.63(0.54, 0.74) | **<0.001** | 0.65(0.55, 0.78) | **<0.001** |
| **Cardiovascular mortality** |  |  |  |  |  |  |  |
| LS - INA | 154/2530 | — |  | — |  | — |  |
| LS - AC | 51/2927 | 0.24(0.16, 0.36) | **<0.001** | 0.4(0.26, 0.61) | **<0.001** | 0.52(0.34, 0.81) | **0.004** |
| LS - IA | 25/1112 | 0.34(0.20, 0.57) | **<0.001** | 0.52(0.31, 0.86) | **0.012** | 0.62(0.38, 1.02) | 0.059 |
| SS - AC | 133/6641 | 0.24(0.18, 0.33) | **<0.001** | 0.34(0.25, 0.47) | **<0.001** | 0.44(0.32, 0.59) | **<0.001** |
| SS - IA | 35/1463 | 0.29(0.20, 0.44) | **<0.001** | 0.32(0.21, 0.49) | **<0.001** | 0.39(0.25, 0.61) | **<0.001** |
| SS - INA | 117/3019 | 0.6(0.45, 0.80) | **<0.001** | 0.58(0.44, 0.76) | **<0.001** | 0.63(0.47, 0.85) | **0.002** |
| Abbreviation: HR: hazard ratio; CI: confidence interval; SB: Sedentary Behavior; PA: Physical Activity; SS - INA (Short - term Sitting, ≤6 h/day & Inactive, PA = 0); SS - IA (Short - term Sitting, ≤6 h/day & Insufficiently active, 0 < PA < 600 MET - min/week); SS - AC (Short - term Sitting, ≤6 h/day & Active, PA ≥ 600 MET - min/week); LS - INA (Long - term Sitting, >6 h/day & Inactive, PA = 0); LS - IA (Long - term Sitting, >6 h/day & Insufficiently active, 0 < PA < 600 MET - min/week); LS - AC (Long - term Sitting, >6 h/day & Active, PA ≥ 600 MET - min/week). Model 1 served as the unadjusted analysis;  Model 2: adjusted for age, sex, and race;  Model 3: fully adjusted model, including additional covariates such as marital status, education attainment, family income, alcohol intake, smoking status, BMI, sitting time, diabetes, and CVD history (congestive heart failure, coronary heart disease, angina pectoris, and stroke). *P-value <0.05 was considered significant. | | | | | | | |

**Supplementary Table 4** Joint association of PA and SB with all-cause, and cardiovascular mortality among individuals with hypertension, stratified by sex.

| **Characteristic** | **Death/No.** | **Model 1** | | **Model 2** | | **Model 3** | |
| --- | --- | --- | --- | --- | --- | --- | --- |
|  |  | **95% CI** | ***p*** | **95% CI** | ***p*** | **95% CI** | ***p*** |
| **Male** |  |  |  |  |  |  |  |
| **PA&SB** | | | | |  | |  |
| **All-cause mortality** | | | | |  | |  |
| LS - INA | 393/1201 | — |  | — |  | — |  |
| LS - AC | 163/1817 | 0.2(0.16, 0.25) | **<0.001** | 0.32(0.26, 0.41) | **<0.001** | 0.32(0.26, 0.41) | **<0.001** |
| LS - IA | 84/562 | 0.4(0.29, 0.57) | **<0.001** | 0.58(0.42, 0.81) | **0.001** | 0.58(0.42, 0.81) | **0.001** |
| SS - AC | 432/4008 | 0.26(0.21, 0.31) | **<0.001** | 0.37(0.31, 0.44) | **<0.001** | 0.37(0.31, 0.44) | **<0.001** |
| SS - IA | 110/608 | 0.42(0.32, 0.55) | **<0.001** | 0.43(0.33, 0.57) | **<0.001** | 0.43(0.33, 0.57) | **<0.001** |
| SS - INA | 294/1285 | 0.67(0.55, 0.83) | **<0.001** | 0.7(0.57, 0.87) | **<0.001** | 0.7(0.57, 0.87) | **<0.001** |
| **Cardiovascular mortality** | |  |  |  |  |  |  |
| LS - INA | 99/1201 | — |  | — |  | — |  |
| LS - AC | 45/1817 | 0.23(0.15, 0.36) | **<0.001** | 0.41(0.27, 0.63) | **<0.001** | 0.41(0.27, 0.63) | **<0.001** |
| LS - IA | 19/562 | 0.33(0.17, 0.64) | **0.001** | 0.51(0.25, 1.01) | 0.053 | 0.51(0.25, 1.01) | 0.053 |
| SS - AC | 120/4008 | 0.27(0.18, 0.39) | **<0.001** | 0.41(0.28, 0.60) | **<0.001** | 0.41(0.28, 0.60) | **<0.001** |
| SS - IA | 20/608 | 0.3(0.18, 0.51) | **<0.001** | 0.32(0.19, 0.53) | **<0.001** | 0.32(0.19, 0.53) | **<0.001** |
| SS - INA | 72/1285 | 0.62(0.43, 0.89) | **0.011** | 0.66(0.46, 0.93) | **0.018** | 0.66(0.46, 0.93) | **0.018** |
| **Female** |  |  |  |  |  |  |  |
| **PA&SB** |  |  |  |  |  |  |  |
| **All-cause mortality** |  |  |  |  |  |  |  |
| LS - INA | 388/1545 | — |  | — |  | — |  |
| LS - AC | 78/1163 | 0.23(0.17, 0.31) | **<0.001** | 0.38(0.30, 0.49) | **<0.001** | 0.46(0.36, 0.58) | **<0.001** |
| LS - IA | 70/585 | 0.42(0.30, 0.60) | **<0.001** | 0.6(0.44, 0.82) | **0.001** | 0.68(0.49, 0.94) | **0.02** |
| SS - AC | 203/2752 | 0.23(0.18, 0.29) | **<0.001** | 0.32(0.26, 0.40) | **<0.001** | 0.39(0.31, 0.48) | **<0.001** |
| SS - IA | 100/886 | 0.31(0.25, 0.39) | **<0.001** | 0.36(0.28, 0.46) | **<0.001** | 0.41(0.33, 0.52) | **<0.001** |
| SS - INA | 305/1867 | 0.6(0.50, 0.72) | **<0.001** | 0.57(0.48, 0.69) | **<0.001** | 0.62(0.51, 0.74) | **<0.001** |
| **Cardiovascular mortality** | |  |  |  |  |  |  |
| LS - INA | 119/1545 | — |  | — |  | — |  |
| LS - AC | 20/1163 | 0.17(0.09, 0.29) | **<0.001** | 0.31(0.19, 0.52) | **<0.001** | 0.4(0.24, 0.67) | **<0.001** |
| LS - IA | 14/585 | 0.28(0.15, 0.50) | **<0.001** | 0.43(0.26, 0.72) | **0.001** | 0.55(0.32, 0.96) | **0.037** |
| SS - AC | 47/2752 | 0.17(0.11, 0.25) | **<0.001** | 0.26(0.18, 0.39) | **<0.001** | 0.35(0.24, 0.50) | **<0.001** |
| SS - IA | 22/886 | 0.22(0.13, 0.38) | **<0.001** | 0.28(0.16, 0.48) | **<0.001** | 0.34(0.19, 0.58) | **<0.001** |
| SS - INA | 71/1867 | 0.47(0.33, 0.67) | **<0.001** | 0.43(0.30, 0.62) | **<0.001** | 0.51(0.35, 0.73) | **<0.001** |
| Abbreviation: HR: hazard ratio; CI: confidence interval; SB: Sedentary Behavior; PA: Physical Activity; SS - INA (Short - term Sitting, ≤6 h/day & Inactive, PA = 0); SS - IA (Short - term Sitting, ≤6 h/day & Insufficiently active, 0 < PA < 600 MET - min/week); SS - AC (Short - term Sitting, ≤6 h/day & Active, PA ≥ 600 MET - min/week); LS - INA (Long - term Sitting, >6 h/day & Inactive, PA = 0); LS - IA (Long - term Sitting, >6 h/day & Insufficiently active, 0 < PA < 600 MET - min/week); LS - AC (Long - term Sitting, >6 h/day & Active, PA ≥ 600 MET - min/week). Model 1 served as the unadjusted analysis;  Model 2: adjusted for age, sex, and race;  Model 3: fully adjusted model, including additional covariates such as marital status, education attainment, family income, alcohol intake, smoking status, BMI, sitting time, diabetes, and CVD history (congestive heart failure, coronary heart disease, angina pectoris, and stroke). *P-value <0.05 was considered significant. | | | | | | | |

**Supplementary Table 5** Joint association of PA and SB with all-cause, and cardiovascular mortality among individuals with hypertension, stratified by age.

| **Characteristic** | **Death/No.** | **Model 1** | | **Model 2** | | **Model 3** | |
| --- | --- | --- | --- | --- | --- | --- | --- |
|  |  | **95% CI** | ***p*** | **95% CI** | ***p*** | **95% CI** | ***p*** |
| **＜60 years** |  |  |  |  |  |  |  |
| **PA&SB** | | | | |  | |  |
| **All-cause mortality** | | | | |  | |  |
| LS - INA | 78/1005 | — |  | — |  | — |  |
| LS - AC | 59/1897 | 0.29(0.19, 0.43) | **<0.001** | 0.26(0.18, 0.39) | **<0.001** | 0.38(0.26, 0.58) | **<0.001** |
| LS - IA | 33/610 | 0.59(0.36, 0.96) | **0.035** | 0.57(0.35, 0.93) | **0.023** | 0.78(0.48, 1.27) | 0.3 |
| SS - AC | 146/3857 | 0.43(0.30, 0.61) | **<0.001** | 0.4(0.29, 0.57) | **<0.001** | 0.47(0.33, 0.68) | **<0.001** |
| SS - IA | 28/653 | 0.39(0.24, 0.64) | **<0.001** | 0.41(0.25, 0.68) | **<0.001** | 0.45(0.28, 0.73) | **0.001** |
| SS - INA | 64/1164 | 0.7(0.44, 1.10) | 0.12 | 0.75(0.48, 1.17) | 0.2 | 0.66(0.42, 1.03) | 0.07 |
| **Cardiovascular mortality** | |  |  |  |  |  |  |
| LS - INA | 23/1005 | — |  | — |  | — |  |
| LS - AC | 17/1897 | 0.38(0.18, 0.79) | **0.01** | 0.35(0.17, 0.72) | **0.004** | 0.63(0.31, 1.30) | 0.2 |
| LS - IA | 5/610 | 0.31(0.17, 0.55) | **<0.001** | 0.3(0.17, 0.54) | **<0.001** | 0.46(0.25, 0.87) | **0.017** |
| SS - AC | 28/3857 | 0.35(0.18, 0.68) | **0.002** | 0.32(0.17, 0.62) | **<0.001** | 0.45(0.21, 0.94) | **0.035** |
| SS - IA | 3/653 | 0.16(0.05, 0.55) | **0.004** | 0.16(0.05, 0.54) | **0.003** | 0.2(0.06, 0.71) | **0.012** |
| SS - INA | 14/1164 | 0.7(0.35, 1.42) | 0.3 | 0.69(0.34, 1.41) | 0.3 | 0.56(0.26, 1.19) | 0.13 |
| **≥60 years** |  |  |  |  |  |  |  |
| **PA&SB** |  |  |  |  |  |  |  |
| **All-cause mortality** |  |  |  |  |  |  |  |
| LS - INA | 703/1741 | — |  | — |  | — |  |
| LS - AC | 182/1083 | 0.34(0.28, 0.41) | **<0.001** | 0.32(0.26, 0.39) | **<0.001** | 0.39(0.32, 0.47) | **<0.001** |
| LS - IA | 121/537 | 0.52(0.41, 0.67) | **<0.001** | 0.52(0.40, 0.67) | **<0.001** | 0.59(0.47, 0.75) | **<0.001** |
| SS - AC | 489/2903 | 0.29(0.25, 0.34) | **<0.001** | 0.28(0.24, 0.33) | **<0.001** | 0.34(0.28, 0.41) | **<0.001** |
| SS - IA | 182/841 | 0.35(0.29, 0.43) | **<0.001** | 0.36(0.30, 0.44) | **<0.001** | 0.41(0.34, 0.50) | **<0.001** |
| SS - INA | 535/1988 | 0.58(0.51, 0.67) | **<0.001** | 0.61(0.53, 0.70) | **<0.001** | 0.64(0.55, 0.73) | **<0.001** |
| **Cardiovascular mortality** | |  |  |  |  |  |  |
| LS - INA | 195/1741 | — |  | — |  | — |  |
| LS - AC | 48/1083 | 0.31(0.21, 0.46) | **<0.001** | 0.29(0.19, 0.43) | **<0.001** | 0.38(0.25, 0.56) | **<0.001** |
| LS - IA | 28/537 | 0.42(0.25, 0.72) | **0.002** | 0.42(0.24, 0.72) | **0.002** | 0.51(0.31, 0.83) | **0.008** |
| SS - AC | 139/2903 | 0.29(0.21, 0.39) | **<0.001** | 0.27(0.20, 0.37) | **<0.001** | 0.35(0.26, 0.47) | **<0.001** |
| SS - IA | 39/841 | 0.27(0.19, 0.41) | **<0.001** | 0.29(0.19, 0.42) | **<0.001** | 0.34(0.23, 0.50) | **<0.001** |
| SS - INA | 129/1988 | 0.48(0.37, 0.63) | **<0.001** | 0.51(0.39, 0.66) | **<0.001** | 0.55(0.42, 0.72) | **<0.001** |
| Abbreviation: HR: hazard ratio; CI: confidence interval; SB: Sedentary Behavior; PA: Physical Activity; SS - INA (Short - term Sitting, ≤6 h/day & Inactive, PA = 0); SS - IA (Short - term Sitting, ≤6 h/day & Insufficiently active, 0 < PA < 600 MET - min/week); SS - AC (Short - term Sitting, ≤6 h/day & Active, PA ≥ 600 MET - min/week); LS - INA (Long - term Sitting, >6 h/day & Inactive, PA = 0); LS - IA (Long - term Sitting, >6 h/day & Insufficiently active, 0 < PA < 600 MET - min/week); LS - AC (Long - term Sitting, >6 h/day & Active, PA ≥ 600 MET - min/week). Model 1 served as the unadjusted analysis;  Model 2: adjusted for age, sex, and race;  Model 3: fully adjusted model, including additional covariates such as marital status, education attainment, family income, alcohol intake, smoking status, BMI, sitting time, diabetes, and CVD history (congestive heart failure, coronary heart disease, angina pectoris, and stroke). *P-value <0.05 was considered significant. | | | | | | | |
